# Adverse Childhood Experiences among a Sample of Youth Living with HIV in the Deep South

**DOI:** 10.3390/ijerph19159740

**Published:** 2022-08-08

**Authors:** Tiffany Chenneville, Hunter Drake, Alexandra Cario, Carina Rodriguez

**Affiliations:** 1 Department of Psychology, University of South Florida, St. Petersburg, FL 33701, USA; 2 Community and Family Health, College of Public Health, University of South Florida, Tampa, FL 33620, USA; 3 Department of Pediatrics, University of South Florida, Tampa, FL 33620, USA

**Keywords:** adverse childhood experiences, ACEs, youth, HIV, YLWH, deep south

## Abstract

The southern region of the United States, often referred to as the Deep South, is disproportionately affected by HIV. In fact, the highest rates of new HIV infections occur in the Deep South. Approximately one in five new HIV infections are among youth. Youth living with HIV (YLWH) have several behavioral health risks, including co-occurring mental health and substance abuse disorders, which negatively affect medication adherence, contribute to less engagement in HIV care, and result in poor health outcomes. Research suggests that adverse childhood experiences (ACEs) contribute to HIV risk behaviors and that people living with HIV may be more vulnerable to the negative health outcomes and adverse effects of stressors. Using existing program evaluation data, we examined data from 41 YLWH aged 17–24 screened for ACEs in an integrated care setting. Most participants were Black/African American young men who identified as homosexual, bisexual, or questioning, and who acquired HIV behaviorally. Approximately, one-third of YLWH screened positive or in the high-risk range on an ACEs screener. Scores fell in the intermediate range for nearly half of the sample. Results did not reveal a significant relationship between ACEs and HIV biological indicators. In this paper, we describe these findings and the importance of incorporating trauma-informed approaches into HIV prevention and treatment programs targeting youth in the Deep South.

## 1. Introduction

The southern region of the United States (US), often referred to as the Deep South, is disproportionately affected by HIV. In fact, the highest rates of new HIV infections occur in the Deep South, and HIV-related deaths are highest in this region of the United States [1]. Yet, compared to other regions, the southern region of the United States receives less funding for HIV prevention and treatment efforts from the federal government and private foundations [1]. HIV-related stigma, racism, and poverty are thought to contribute to high rates of HIV and poor outcomes for people living with HIV (PLWH) in the Deep South [1,2].

In the United States, approximately 21% of new HIV infections are among youth ages 13 to 24 [3]. Gay and bisexual men account for the highest number of new HIV diagnoses among youth, and Black/African American youth are disproportionately affected by HIV [3]. Compared to all other age groups, youth are the least likely to know their HIV status [3]. In fact, it is estimated that 45% of youth are unaware of their HIV status compared to 14% of all people living with HIV [3]. Similarly, YLWH are the least likely to be virally suppressed [3]. These outcomes are thought to be associated with unique social and economic challenges facing youth, along with low rates of HIV testing and pre-exposure prophylaxis (PrEP) use, and high rates of other sexually transmitted infections, among this population [3]. Further, evidence suggests that YLWH often have co-occurring mental health and substance abuse disorders [4]. These co-occurring diagnoses can negatively affect medication adherence, contribute to less engagement in HIV care, and result in poor health outcomes [5].

Research suggests that adverse childhood experiences (ACEs) contribute to HIV risk behaviors [6,7]. ACE categories include abuse and neglect as well as other forms of household dysfunction [8]. In a study of adult men who have sex with men (MSM), nearly 80% reported exposure to one or more ACEs [9]. Those who endorsed ACEs were more likely to report illicit substance use and condomless anal intercourse [9]. There also is evidence that ACEs can affect the immune system and are related to morbidity in adulthood [7,10], which is of particular concern for PLWH. PLWH may be more vulnerable to the negative health outcomes and adverse effects of stressors of ACEs [11]. In a southeastern urban clinic in the United States, ACEs were associated with unsuppressed viral load and missed appointments for PLWH [12].

Unfortunately, much of the research on the impact of ACEs on HIV prevention and treatment has not focused on youth. Among the few studies that have been conducted using youth samples, most were conducted in low to middle income countries [13,14,15]. To the authors’ knowledge, there are no studies on ACEs among youth living with HIV (YLWH) in the Deep South. To address this important gap in the literature, the purpose of this exploratory study was to assess ACEs among a sample of YLWH receiving treatment in an integrated care setting in the southeastern United States, to examine differences in ACEs based on demographic variables, and to explore the relationship between ACEs, biological indicators, and mental health, namely mental illness diagnosis and substance use.

## 2. Materials and Methods

### 2.1. Participants and Procedures

This study used existing de-identified data gathered to improve patient services in an integrated care setting specializing in the treatment of pediatric and adolescent HIV in the southeastern United States, the heart of the Deep South. The program serves children and youth from birth to age 25 with perinatally and behaviorally-acquired HIV although most patients present with behaviorally-acquired HIV. Consistent with data on the high rates of HIV among racial and ethnic minority groups in the Deep South [1,3], most patients are Black or African American and non-Hispanic young men. In the United States, race is distinguished from ethnicity. *Black or African American* is a racial category that encompasses all Black racial groups while *Hispanic* and *non-Hispanic* are ethnic categories.

Data on adverse childhood experiences (ACEs) were collected from a subset of patients from January to December 2019 and extracted based on an archival review of an existing program evaluation database. Specifically, two case managers administered the ACEs screener to established patients aged 18 and older who were seen in the clinic during 2019. The age limit was determined by the ACEs questionnaire (see below), which instructs participants to report experiences during the first 18 years of their life. An exception was made for one 17-year-old who was turning 18 the day after the administration of the ACEs screener. Since this study relied on anonymous program evaluation data, it was exempt from institutional review board approval.

### 2.2. Measures

The following demographic data were recorded: age, race, ethnicity, gender identity, sexual orientation, and mode of HIV transmission. The 10-item Adverse Childhood Experiences (ACEs) questionnaire [8] was administered as a screening instrument to assess childhood trauma among YLWH. Individual items on the ACEs questionnaire assess for physical abuse, verbal abuse, sexual abuse, physical neglect, emotional neglect, parental separation or divorce, interpersonal violence in the home, problematic substance use by a household member, mental illness of a household member, and prison experience of a household member during childhood (prior to age 18). Example items include *Was a household member depressed or mentally ill or did a household member attempt suicide?* and *Did a household member go to prison?* Response options are *yes* or *no*. Each item marked *yes* is assigned one point. Each item marked *no* is assigned a score of 0. Scores are then summed for a total score. A total score of 0 is considered low risk. Scores between 1–3 are considered intermediate risk. A score of 4 or higher is considered high risk. Scoring criteria and classifications are based on Felitti et al.’s original ACEs study [8]. For this study, a positive screener was indicated by a score of 4 or higher.

### 2.3. Data Analysis

Descriptive statistics were used to characterize the sample and scores on the ACEs screener. One-tailed Pearson correlations were run to investigate the relationship between individual items on the ACE screener and substance use, the presence of mental illness diagnoses, as well as CD4 count and viral load biostatistics. One-way ANOVAs were run to test for differences in ACE scores by race, ethnicity, gender identity, sex at birth, education, employment, living situation, income, sexual orientation, or mode of HIV transmission. A simple linear regression was run to test age as a significant predictor of ACE scores. Analyses were performed using IBM SPSS Statistics for Windows, Version 28.

## 3. Results

### 3.1. Participants

Participants were 41 YLWH ages 17–24 (*M* = 20.85, *SD* = 1.89). Most respondents were non-Hispanic (*n* = 36, 87.80%) Black or African American (*n* = 29, 70.73%) boys or young men (*n* = 30, 73.17%) who acquired HIV behaviorally (*n* = 28, 68.3%). There was an even distribution of participants who identified as homosexual (*n* = 18, 43.90%) and heterosexual (*n* = 18, 43.90%) with the remaining participants identifying as bisexual (*n* = 4, 9.8%) or questioning (*n* = 1, 2.44%). The use of alcohol (*n* = 17, 41.5%), marijuana (*n* = 19, 46.3%), or other illicit drugs (*n* = 2, 4.9%) was reported by a majority (*n* = 24, 58.5%) of participants. Mental illness diagnoses were documented among some participants (*n* = 8, 19.5%) to include: Autism Spectrum Disorder (*n* = 1, 2.4%), Generalized Anxiety Disorder (*n* = 1, 2.4%), Major Depressive Disorder (*n* = 2, 4.9%), Attention Deficit Hyperactivity Disorder (*n* = 5, 12.2%), and Bipolar Disorder (*n* = 2, 4.9%). The CD4 count among participants ranged from 100 to 1393 with 75.6% (*n* = 31) having a CD4 count at or above 500, which is a good prognostic indicator. The viral load among participants range from 20 to 65,398 with 82.9% (*n* = 34) having a viral load equal to or less than 50 copies, which is a good prognostic indicator. See Table 1 for a complete account of demographic characteristics for the sample.

### 3.2. Adverse Childhood Experiences (ACEs)

Among the 41 cases, there were 14 (34.15%) positive screeners with four or more endorsements on the 10-item ACE screener. Approximately half of participants’ ACE scores (*n* = 20, 49.8%) were below the cut point for a positive screen but still indicated intermediate risk. There were 34 (82.9%) participants who endorsed at least one of the 10 ACE items; only 7 participants (17.1%) did not endorse any ACE items at all. The most frequently endorsed ACE items were 1, *Did a parent or other adult in the household often swear at you, insult you, put you down, or humiliate you, OR act in a way that made you afraid you would be physical hurt?* (*n* = 20, 48.8%); 2, *Did a parent or other adult in the household often push, grab, slap, or throw something at you, OR ever hit you so hard that you had marks or were injured?,* (*n* = 15, 36.6%); Item 4, *Did you often feel that no one in your family loved you or thought you were important or special?,* (*n* = 16, 39.0%); and Item 6, *Were your parents ever separated or divorced?* (*n* = 26, 63.4%). Descriptions and frequencies of endorsement for each individual ACE item can be found in Table 2.

### 3.3. Differences between ACEs Based on Demographic Variables

One-way ANOVAs revealed no significant differences in ACE total scores based on race, ethnicity, gender identity, sex at birth, education, employment, living situation, income, sexual orientation, or mode of HIV transmission. Linear regression revealed that age was not a significant predictor of ACE total scores (*F*_(1,39)_ = 0.353, *p* = 0.556; *β*_Age_ = −0.120, *SE* = 0.202, *t* = −0.594, *p =* 0.202). See Table 3 for detailed statistics.

### 3.4. Relationship between ACEs and Biological Indicators

Pearson correlation analysis revealed no significant relationship between individual ACE items and CD4 counts or viral load. However, the presence of a mental illness diagnosis had a weak negative relationship with CD4 counts (*r* = −0.279, *p* = 0.038). See Table 4 for correlation details.

### 3.5. Relationship between ACEs and Substance Use

Pearson correlation analyses revealed significant relationships between three ACE items and substance use. Item 4, “Did you often feel that no one in your family loved you or thought you were important or special OR your family didn’t look out for each other, feel close to each other, or support each other?”, showed a weak positive relationship with the current use of other substances (r = 0.283, *p* = 0.036). Item 8, “Did you live with anyone who was a problem drinker or alcoholic or who used street drugs?”, showed a weak negative relationship with the current use of marijuana (r = −0.300, *p* = 0.028). Item 10, “Did a household member go to prison?”, revealed a weak negative relationship with the current use of alcohol (r = −0.329, *p* = 0.018). Additionally, the current use of alcohol was positively, moderately related to the current use of marijuana (r = 0.409, *p* = 0.004). See Table 4 for complete correlation details.

## 4. Discussion

Findings from this study revealed that nearly one third of the YLWH in our sample screened for ACEs scored positive, having endorsed four or more ACE categories. Approximately half of the YLWH in our sample scored in the intermediate range. Combined, these findings suggest very few YLWH scored in the low-risk range. Given evidence that ACEs can affect the immune system, disease, morbidity, and mental health [10,16], this is particularly concerning because YLWH already are less likely to be virally suppressed [3] and are likely to have co-occurring mental health and substance use disorders that negatively affect medication adherence, contribute to less engagement in HIV care, and result in poor health outcomes [5]. This is also concerning because ACEs are associated with risky behaviors [10], which may increase the likelihood of secondary transmission of HIV among YLWH. The context of the Deep South heightens concerns. Although over half the Black population in the United States lives in the South [17], owing in large part to America’s shameful history of slavery and the concentration of slaves in southern states, racism and resulting health disparities are disproportionately high in this region of the United States. There also are high rates of HIV-related stigma and poverty in this region, which contribute to poor outcomes for PLWH [1,2], including youth, and are made worse by a lack of federal and private funding for HIV prevention and treatment [1].

Unlike Giano et al. [18] who found ACEs were higher among women, Black and Hispanic individuals, and those who identified as sexual minorities in a large sample of adults across 34 states in the United States, there were no differences in the present study in ACEs based on race, ethnicity, gender identity, sex at birth, or sexual orientation in our sample. This is surprising given the prevalence of racism and homophobic views in the Deep South. Our findings also did not reveal a significant difference in ACEs based on education, employment, living situation, or income.

Results from this study did not reveal differences in ACEs based on mode of transmission—perinatal versus behavioral. This might be surprising given that in utero exposure to HIV and/or antiretrovirals (based on maternal use during pregnancy) may be considered an adverse event during a sensitive developmental period, which is the case for youth with perinatally-acquired HIV. Indeed, findings from a systematic review of the literature provided some evidence of neurobehavioral differences between youth exposed to HIV and antiretrovirals in utero and those not exposed [19]. However, as authors of the systematic review note, data are limited to substantially support the claim that youth exposed in utero to HIV and/or antiretrovirals have worse neurodevelopmental outcomes [19].

Current findings also did not reveal a relationship between ACEs and biological indicators (i.e., T-cell and viral load), which is inconsistent with a study on PLWH in a large urban HIV clinic for adults in the southeastern United States, which found that ACEs were associated with unsuppressed viral load [12]. Our findings are difficult to explain but may be attributed, in part, to the intersection of age, time since diagnosis, and mode of transmission. Most participants in our study contracted HIV behaviorally, which means fewer years living with HIV compared to youth with perinatally-acquired HIV or adults. As a result, the YLWH in our sample may have been less affected by HIV treatment fatigue [20]. Some research also suggests that youth with behaviorally-acquired HIV have fewer barriers to medication adherence than youth with perinatally-acquired HIV [21]. Therefore, it may be that antiretroviral adherence among YLWH, particularly those with behaviorally-acquired HIV, is not negatively affected by ACEs but may be affected with age (into middle and older adulthood).

Albeit weak, current findings did reveal a relationship between some ACEs and substance use. This is consistent with findings from a systematic review, which documented that ACEs are common among people and correlated with substance use diagnoses [22]. Findings did not reveal a relationship between ACEs and mental illness diagnoses, which is inconsistent with findings from a review of the literature suggesting that ACEs are a primary cause of mental illness [23]. In our sample, only 20% of YLWH had been diagnosed with a mental illness, which may be a function of age and mental health care access. That is, symptoms of mental illness may still be emerging during youth. This, combined with limited access to mental health care in the Deep South [24], may have resulted in the underdiagnosis of mental illness among the YLWH in our sample.

Research suggests that social support may serve as a protective factor against ACEs in the general population. For example, studies have shown that social support may act as a buffer to ACEs in relation to symptoms of depression [25,26] and anxiety [27]. It stands to reason that social support would also serve as a protective factor against ACEs among YLWH. However, social support was not examined in this study, and the existing literature on ACEs and HIV focuses primarily on the role of ACEs on HIV risk behaviors.

Current findings contribute to the growing body of literature on the impact of ACEs on HIV prevention and treatment which, to date, has focused primarily on adult populations. This study also contributes to our knowledge about ACEs among YLWH in the Deep South. A better understanding of the role of early adverse experiences may help improve the continuum of care for people living with HIV [11], including youth living in the Deep South, a region with well documented health disparities and that is in desperate need expanded healthcare services.

These findings support the need for a trauma-informed approach to HIV prevention and treatment [6] (Fang, Chuang, and Lee, 2016), especially given the historical trauma associated with anti-Black racism [28], which is particularly prevalent in the Deep South. Myers et al. [29] developed a trauma-informed substance use and sexual risk reduction intervention for youth that involved six group sessions. Although the intervention was developed for young women in South Africa, it could be adapted for other populations, including youth in the Deep South. Indeed, Sales et al. [30] advocated for the testing of trauma-informed care for groups with high rates of HIV and trauma such as men who have sex with men and transgendered populations.

In addition to the need for trauma-informed care, a life course approach to HIV prevention and treatment has been recommended as a means by which to address ACEs among people at risk for or living with HIV [31]. This approach acknowledges that key stages in life across the lifespan—from birth to death—pose unique challenges and opportunities for disease prevention and treatment [32]. Transitions in HIV care, from pediatric to adult care, exemplify the challenges and opportunities highlighted by the life course approach to disease management. Indeed, this transition represents a key stage in the life of YLWH and may be an important time for intervention.

Limitations include the use of a small sample from one clinic in the southeastern United States, which affects generalizability. Further, small sample sizes may have limited the power to detect differences between groups. The use of program evaluation data also was a limitation in that it required retrospective analysis and also restricted the data available for analysis including data on potential confounding variables (e.g., social support). The absence of a control group further limits the interpretation of findings. Finally, while the use of the original 10-item ACEs instrument [8] may be considered a strength in some aspects, it also may be considered a weakness in that many other adversities exist, which were not assessed in this study. Despite limitations, findings have important implications for HIV healthcare providers working with youth.

## 5. Conclusions

Findings reveal high rates of ACEs among a sample of YLWH living in the Deep South. Although current findings did not reveal a relationship between ACEs and biological indicators, there was a relationship between ACEs and mental illness diagnosis and substance use. Given that mental illness and substance use may negatively affect HIV disease management, findings support the need for trauma-informed approaches to meet the needs of YLWH in the Deep South. Trauma informed interventions that incorporate life course approaches may be particularly important to combat the negative impact of ACEs on health for YLWH as they transition into adulthood. Future research is needed with larger samples and a control group to further explore the prevalence and impact of ACEs among YLWH.

## Figures and Tables

**Table 1:** Demographics.

*Sample Characteristics (n = 41)*					
		***n***	**%**		
**Demographics** Race					
	Black or African American	29	70.7%		
	White	8	19.5%		
	Asian	2	4.9%		
	Other	2	4.9%		
Ethnicity					
	Hispanic	5	12.2%		
	Non-Hispanic	36	87.8%		
Mode of Transmission					
	Perinatal	13	31.7%		
	Behavioral	28	68.3%		
Sexual Orientation					
	Heterosexual	18	43.9%		
	Homosexual	18	43.9%		
	Bisexual	4	9.8%		
	Questioning	1	2.4%		
Gender Identity					
	Male	27	65.9%		
	Female	11	26.8%		
	Transgender Female	3	7.3%		
Sex at Birth					
	Male	30	73.2%		
	Female	11	26.8%		
**Socioeconomic** **Characteristics** Education					
	Middle School	1	2.4%		
	Some High School	8	19.5%		
	High School Degree	12	29.3%		
	High School Equivalency (GED)	4	9.8%		
*Sample Characteristics (n = 41)*					
		***n***	**%**		
	Some College (No Degree)	13	31.7%		
	Associate’s Degree	2	4.9%		
	Bachelor’s Degree	1	2.4%		
Employment					
	Unemployed, Not Looking	1	2.4%		
	Unemployed, Looking	11	26.8%		
	Employed Part-Time	7	17.1%		
	Employed Full-Time	17	41.5%		
	Self-Employed	1	2.4%		
	Student	4	9.8%		
Living Situation					
	Alone	6	14.6%		
	Spouse	1	2.4%		
	Parents	16	39.0%		
	Siblings	1	2.4%		
	Other Relatives	3	7.3%		
	Roommate(s) or Friend(s)	5	12.2%		
	Significant Other/Partner	6	14.6%		
	Other Residents (Group Living Situation)	3	7.3%		
Income					
	<USD 12,000	18	43.9%		
	USD 12,000–USD 20,000	5	12.2%		
	USD 21,000–USD 30,000	7	17.1%		
	USD 31,000–USD 40,000	7	17.1%		
	USD 41,000–USD 50,000	2	4.9%		
	>USD 51,000	1	2.4%		
	Did Not Wish to Answer	1	2.4%		
*Sample Characteristics (n = 41)*					
**Mental Health** **Characteristics** Substance Use					
		**Yes**		**No**	
		***n***	**%**	***n***	**%**
	Reported Substance Use of Any Kind	24	58.5%	17	41.5%
	Alcohol Use	17	41.5%	24	58.5%
	Marijuana Use	19	46.3%	22	53.7%
	Other Illicit Substance Use	2	4.9%	39	95.1%
Mental Illness Diagnosis					
	Any Mental Illness Diagnosis	8	19.5%	33	80.5%
	Autism Spectrum Disorder	1	2.4%	40	97.6%
	Generalized Anxiety Disorder	1	2.4%	40	97.6%
	Major Depressive Disorder	2	4.9%	39	95.1%
	Attention Deficit Hyperactivity Disorder	5	12.2%	36	87.8%
	Bipolar Disorder	2	4.9%	39	95.1%

**Table 2:** Endorsement of individual ACE items.

*Sample Size (n = 41)*				
**Item #**	**Description**	**Response Options**	***n Endorsed***	**% of *n* = 41 Participants**
ACE 1	Did a parent or other adult in the household often… Swear at you, insult you, put you down, or humiliate you? OR Act in a way that made you afraid that you might be physically hurt?	YES (1)/NO (0)	20	48.8%
ACE 2	Did a parent or other adult in the household often… Push, grab, slap, or throw something at you? OR Ever hit you so hard that you had marks or were injured?	YES (1)/NO (0)	15	36.6%
ACE 3	Did an adult or person at least 5 years older than you ever… Touch or fondle you or have you touch their body in a sexual way? OR Try to or actually have oral, anal, or vaginal sex with you?	YES (1)/NO (0)	9	22.0%
ACE 4	Did you often feel that... No one in your family loved you or thought you were important or special OR your family didn’t look out for each other, feel close to each other, or support each other?	YES (1)/NO (0)	16	39.0%
ACE 5	Did you often feel that… You didn’t have enough to eat, had to wear dirty clothes, and had no one to protect you? OR Your parents were too drunk or high to take care of you or take you to the doctor if you needed it?	YES (1)/NO (0)	5	12.2%
ACE 6	Were your parents ever separated or divorced?	YES (1)/NO (0)	26	63.4%
ACE 7	Was your mother or stepmother: Often pushed, grabbed, slapped, or had something thrown at her? OR Sometimes or often kicked, bitten, hit with a fist, or hit with something hard? OR Ever repeatedly hit over at least a few minutes or threatened with a gun or knife?	YES (1)/NO (0)	4	9.8%
ACE 8	Did you live with anyone who was a problem drinker or alcoholic or who used street drugs?	YES (1)/NO (0)	10	24.4%
ACE 9	Was a household member depressed or mentally ill or did a household member attempt suicide?	YES (1)/NO (0)	7	17.1%
ACE 10	Did a household member go to prison?	YES (1)/NO (0)	10	24.4%

**Table 3:** Group differences by demographics.

*Sample Size (n = 41)*						
	***F***	***df_1_,df_2_***	***p***	**η^2^**	**η^2^ CI**	
					**Lower**	**Upper**
Race	2.714	3,37	0.059	0.180	0.000	0.344
Ethnicity	1.510	1,39	0.226	0.037	0.000	0.200
Mode of Transmission	0.103	1,39	0.750	0.003	0.000	0.104
Sexual Orientation	1.542	3,37	0.220	0.111	0.000	0.262
Gender Identity	0.311	2,38	0.734	0.016	0.000	0.119
Sex at Birth	0.297	1,39	0.589	0.008	0.000	0.131
Education	0.911	6,34	0.499	0.138	0.000	0.229
Employment	1.655	5,35	0.171	0.191	0.000	0.319
Living Situation	1.683	7,33	0.148	0.263	0.000	0.362
Income ^1^	0.473	5,34	0.794	0.065	0.000	0.133
	*F*	*p*	*β*	*SE*	*t*	*p*
Age	0.353	0.556	−0.120	0.202	0.594	0.202

^1^ One participant declined to respond to the item asking for income range.

**Table 4:** ACE item correlations.

*Sample Size (n = 41)*												
	**Alcohol Use**		**Marijuana Use**		**Use of Other Substances**		**Living with Mental Illness**		**CD4 (T-Cell) Count**		**Viral Load**	
	*r*	*p*	*r*	*p*	*r*	*p*	*r*	*p*	*r*	*p*	*r*	*p*
ACE 1	0.07	0.332	−0.03	0.435	0.01	0.486	−0.05	0.388	−0.10	0.263	−0.02	0.457
ACE 2	0.18	0.126	0.00	0.488	0.06	0.348	−0.04	0.412	0.00	0.493	0.04	0.405
ACE 3	0.03	0.421	0.10	0.271	0.15	0.169	0.15	0.181	−0.04	0.401	0.12	0.221
ACE 4	−0.06	0.345	−0.04	0.398	0.283 *	0.036	0.18	0.130	0.05	0.369	0.03	0.433
ACE 5	−0.01	0.473	−0.20	0.109	−0.08	0.300	0.16	0.155	−0.18	0.136	0.22	0.080
ACE 6	0.23	0.076	0.20	0.107	−0.06	0.348	0.04	0.412	0.06	0.345	−0.09	0.291
ACE 7	0.06	0.362	0.02	0.440	−0.07	0.322	0.22	0.081	0.00	0.491	−0.07	0.323
ACE 8	−0.13	0.205	−0.300 *	0.028	0.14	0.200	0.11	0.246	−0.24	0.068	0.11	0.250
ACE 9	0.01	0.468	0.10	0.270	0.20	0.107	0.23	0.075	0.06	0.354	0.17	0.150
ACE 10	−0.329 *	0.018	0.04	0.398	0.14	0.200	−0.16	0.153	0.00	0.494	0.11	0.253
Alcohol Use	--		0.409 **	0.004	0.04	0.404	−0.09	0.293	0.02	0.462	0.06	0.349
Marijuana Use			--		0.24	0.062	0.10	0.271	0.02	0.439	0.04	0.402
Use of Other Substances					--		−0.12	0.227	0.04	0.396	−0.05	0.375
Living with Mental Illness							--		−0.279 *	0.038	−0.12	0.228
CD4									--		−0.19	0.111

** Correlation is significant at the 0.01 level (1-tailed). * Correlation is significant at the 0.05 level (1-tailed).

## Data Availability

The data presented in this study are available on request from the corresponding author. The data are not publicly available due to the fact that this study relied on existing program evaluation data.
